# Evaluating the potential of hyperpolarised [1-^13^C] L-lactate as a neuroprotectant metabolic biosensor for stroke

**DOI:** 10.1038/s41598-020-62319-x

**Published:** 2020-03-26

**Authors:** Jean-Noël Hyacinthe, Lara Buscemi, Thanh Phong Lê, Mario Lepore, Lorenz Hirt, Mor Mishkovsky

**Affiliations:** 1 Geneva School of Health Sciences, HES-SO University of Applied Sciences and Arts Western Switzerland, Geneva, Switzerland; 2 Image Guided Intervention Laboratory, Faculty of Medicine, University of Geneva, Geneva, Switzerland; 3 Department of Clinical Neurosciences, Centre Hospitalier Universitaire Vaudois, Lausanne, Switzerland; 4 Laboratory of Functional and Metabolic Imaging, École polytechnique fédérale de Lausanne (EPFL), Lausanne, Switzerland; 5 Centre d’Imagerie Biomédicale (CIBM), École polytechnique fédérale de Lausanne (EPFL), Lausanne, Switzerland

**Keywords:** Metabolic pathways, Neuroscience, Translational research

## Abstract

Cerebral metabolism, which can be monitored by magnetic resonance spectroscopy (MRS), changes rapidly after brain ischaemic injury. Hyperpolarisation techniques boost ^13^C MRS sensitivity by several orders of magnitude, thereby enabling *in vivo* monitoring of biochemical transformations of hyperpolarised (HP) ^13^C-labelled precursors with a time resolution of seconds. The exogenous administration of the metabolite L-lactate was shown to decrease lesion size and ameliorate neurological outcome in preclinical studies in rodent stroke models, as well as influencing brain metabolism in clinical pilot studies of acute brain injury patients. The aim of this study was to demonstrate the feasibility of measuring HP [1-^13^C] L-lactate metabolism in real-time in the mouse brain after ischaemic stroke when administered after reperfusion at a therapeutic dose. We showed a rapid, time-after-reperfusion-dependent conversion of [1-^13^C] L-lactate to [1-^13^C] pyruvate and [^13^C] bicarbonate that brings new insights into the neuroprotection mechanism of L-lactate. Moreover, this study paves the way for the use of HP [1-^13^C] L-lactate as a sensitive molecular-imaging biosensor in ischaemic stroke patients after endovascular clot removal.

## Introduction

Stroke is the second most common cause of death and the third leading cause of disability worldwide^1^. Approximately 85% of strokes are of the ischaemic subtype that can be treated by restoring blood flow to the ischaemic brain through thrombolysis or thrombectomy within a relatively narrow time window of 4.5–7.3 hours after ischemic onset^2–4^. Interestingly, the evolution of ischaemic damage can vary between patients and careful patient selection based on imaging properties of the ischaemic brain allows performing thrombolysis up to 9 hours after ischemia onset and thrombectomy up to 24 hours with a significant improvement in the outcome^5^.

Thanks to recent advances^5^, the number of stroke patients receiving treatment increased in the past years (now about 15–20% in Switzerland), but even for treated patients good outcome is not guaranteed. In addition to timely clot-removal interventions, neuroprotective strategies applied in the acute phase of ischaemic stroke could improve patient outcome by enhancing the recovery of brain cells not yet irreversibly damaged or by promoting the brain’s endogenous self-repair mechanisms. Nonetheless, despite the many different neuroprotective approaches proposed from preclinical results, so far none were shown to be effective at a clinical level^6,7^.

It is well established that large amounts of the metabolite lactate are produced in brain areas subjected to hypoxia due to reduced blood supply^8,9^. Once considered an end product of metabolism, increasing evidence supports a role of lactate as an energy substrate for the brain^10,11^ leading to the hypothesis that elevated lactate concentrations after cerebral ischaemia can be used to fuel the brain. Several *in vivo* studies have emphasised the need of lactate to sustain neuronal recovery directly after ischaemia^12–14^. Interestingly, it has been shown that exogenous lactate administration after transient cerebral ischaemia at reperfusion protects against ischemia-induced cell death and disability^15,16^. A dual mode of action was suggested for lactate to provide neuroprotection by constituting an energy substrate transported by monocarboxylate transporters (MCTs) to deprived neurons, or by signalling via the hydroxycarboxylic acid receptor 1 (HCAR1), thus involving both metabolism and signalling^17^. Furthermore, in pilot clinical studies, lactate was safely administered to acute brain injury patients and proved to be beneficial against intracranial hypertension and improved cerebral glucose availability^18,19^.

Magnetic resonance spectroscopy (MRS) techniques provide tools to study cerebral metabolism non-invasively. The evolution of the neurochemical profile composed of the endogenous molecules detected by ^1^H MRS after cerebral ischaemia-reperfusion^8,9,20,21^ illustrates the impact of ischaemia on brain metabolism. MRS can also be used to track metabolism of exogenously infused non-hyperpolarised (i.e. thermally polarised) ^13^C-labelled metabolic tracers using ^13^C MRS, but its low sensitivity limits clinical application. The recent development of hyperpolarised (HP) ^13^C MRS by dissolution dynamic nuclear polarisation (dDNP)^22^ enables boosting MR sensitivity and monitoring brain metabolism *in vivo* in real time in preclinical studies^23–27^ and clinical settings^28–30^. In the context of ischaemic stroke, it was recently demonstrated that molecular imaging of HP [1-^13^C] pyruvate could highlight differences between the penumbra and intact remote regions^31^. Lactate is among the many endogenous molecules that can be polarised^32–36^ and given that the plasma concentration of endogenous lactate is rather high, it can be safely administered at the typical millimolar concentrations necessary for both HP ^13^C MRS measurements^33–36^ and neuroprotection^16^.

The aim of this study was to demonstrate the feasibility of measuring HP [1-^13^C] lactate metabolism in the mouse brain when administered at a therapeutic dose after ischaemic stroke at different reperfusion time points to evaluate the potential of HP lactate as a theranostic biosensor for stroke. Notably, kinetics of HP lactate metabolism may help to shed light on the puzzling dual mechanism of lactate neuroprotection.

## Materials and Methods

### Animal experimentation

All experimental procedures involving mice were approved by the regulatory body of the Canton Vaud, Switzerland (Service de la consommation et des affaires vétérinaires) license number VD2017.5, and all experiments were conducted according to Federal and local ethical guidelines and complied with the ARRIVE guidelines. Male C57BL/6 J mice (n = 34, 7–9 weeks of age, body weight 25.71 ± 2.14 g, Charles River, France) were given free access to food and water and were maintained in a 12 h light-dark cycle in a temperature- and humidity-controlled animal facility.

### Transient middle cerebral artery occlusion (MCAO)

Mice were anaesthetised with isoflurane (1.5–2.0% in 60% oxygen) using a facemask. Regional cerebral blood flow (rCBF) was measured throughout the operation by laser-Doppler flowmetry (Perimed AB, Sweden) with a flexible probe fixed on the skull at 1 mm posterior and 6 mm lateral from bregma. Transient focal cerebral ischaemia was induced by occlusion of the left middle cerebral artery (MCA) with an intra-luminal suture as described previously^17,37^. Briefly, the left common carotid artery and the left external carotid artery were surgically exposed and ligated following a ventral midline neck incision and ischaemia was induced by inserting a silicone-coated nylon monofilament (Doccol Corp, USA) through the common carotid artery into the internal carotid artery. rCBF was maintained below 20% of the baseline for 30 minutes, after which the occluding filament was withdrawn to allow reperfusion. Occlusion was considered successful if rCBF decreased by at least 80% of the start value and reperfusion if the rCBF rose above 50% of baseline within 10 min after filament removal. 5 out of the 34 mice that underwent the surgical procedure were not included in the study, as one did not reach the 80% reduction during occlusion and 4 mice did not reperfuse to 50% of the starting rCBF. Sham operated mice underwent a similar surgical procedure but without MCA occlusion. In order to minimise anaesthesia duration, the femoral vein was catheterised during the 30-minute ischaemia to allow subsequent intravenous (i.v.) HP [1-^13^C] lactate injection.

### Hyperpolarisation

HP [1-^13^C] lactate was prepared as previously described^34^. Briefly, frozen beads containing sodium [1-^13^C] L-lactate solution (45–55% (w/w) in H_2_O, Sigma Aldrich) and d_8_-glycerol (Sigma Aldrich) were mixed 1:1 (w/w). The sample was dynamically polarised using TEMPOL radical (58 mM, Sigma Aldrich) as polarising agent on a custom-designed 7 T DNP polariser operating at 197 GHz/1.00 ± 0.05 K^38^ for 2 h, then rapidly dissolved in 5 mL of superheated D_2_O and transferred within 2 s into the separator/infusion pump^39^, which was positioned inside the magnet bore. The [1-^13^C] lactate polarisation measured at the separator was 15.6 ± 2.0% (n = 5). A bolus of the solution was automatically infused over 3 s through the catheterised mouse femoral vein as previously described^40^.

### Magnetic resonance measurements

We performed all measurements on a Varian INOVA spectrometer (Varian, Palo Alto, CA, USA) interfaced to a 31 cm horizontal-bore actively shielded 9.4 T magnet (Magnex Scientific, Abingdon, UK).

#### Hyperpolarised ^13^C MRS

Directly after reperfusion, animals were placed into the MR scanner. We performed ^13^C MR measurements using a home-built ^1^H-quadrature/^13^C-single loop surface coil that we placed on top of the mouse’s head. The sensitivity profile of the ^13^C-single loop surface coil limits the signal detection to the mouse head. Coil specifications are presented in the Supporting Information (Fig. S1). B_0_ inhomogeneity was corrected using the FASTESTMAP algorithm^41^. Anatomical T_2_ weighted (T_2W_) images were acquired (fast spin echo imaging) before i.v. bolus infusion of the HP solution (either 325 or 350 μL, 90 mM HP [1-^13^C] lactate solution and a dead volume of either 125 μL or 350 μL respectively, note that in earlier measurements we injected the larger dead volume and then improved our system to reduce it) by the automated protocol^40^. The chosen lactate dose (1.16 mmol/kg) was based on the optimal therapeutic dose used in Berthet *et al*.^16^ and resulted in a [1-^13^C] lactate concentration in blood immediately after injection of 12.9 ± 0.9 mM. HP solutions were injected 1 h or 2 h post-reperfusion after MCAO surgery and 1 h post-surgery in sham operated animals (n = 5 each, 15 mice in total). A series of pulse-acquire (30°, BIR-4 pulse^42^) spectra was triggered 1.5 s post-injection and repeated every 3 s for 3 min.

The area under the curve (AUC) of the metabolites was calculated using VNMRJ software by integrating the ^13^C MRS spectra after phase and baseline correction. The peak areas of [1-^13^C] lactate, [1-^13^C] pyruvate, and [^13^C] bicarbonate were quantified, averaged over 10 acquisitions (sum of spectra 3–13, corresponding to a total acquisition time of 37.5 s), and used to compute the pyruvate-to-lactate (PLR) and bicarbonate-to-lactate (BLR) ratios. To reduce the variability between animals, data was corrected by scaling it to the HP [1-^13^C] lactate concentration in blood immediately after the injection, namely by multiplying the number of moles of [1-^13^C] lactate injected (i.e. 90 mM multiplied by either 325 or 350 μL) and dividing by the animal’s blood volume plus the total injected volume resulting in corrected ratios cPLR and cBLR respectively. The blood volume of the individual animal was estimated as 7% of the body weight^43^.

#### Proton MRS

Proton spectra were acquired on a different set of animals after MCAO surgery (n = 3) and sham operated animals (n = 3) both without lactate injection. We performed the measurements using a home-built ^1^H-quadrature surface coil located on top of the mouse’s head. Quickly after completion of the 30 min MCAO with successful reperfusion, animals were placed carefully into the MR scanner. T_2W_ images were acquired and B_0_ inhomogeneity was corrected at the voxel located at the striatum where the lesion is expected to form (2 × 1.8 × 2 mm^3^, 7.2 μL). A series of ^1^H MRS spectra were acquired using SPECIAL pulse sequences^44^ (TE/TR = 2.8/4000 ms, 200 ms acquisition time in 10 × 16 scans) and repeated continuously until about 200 min post-reperfusion. Metabolite concentrations were calculated using LCModel-based fitting routine^45^.

### Immunostaining

To assess variation in monocarboxylate transporter (MCT) distribution and expression following 30 min MCAO at the injection time points (i.e. 1 h and 2 h post-reperfusion), we performed immunostaining using MCT1, MCT2 and MCT4 antibodies. Eight male C57BL/6 J mice (sham, n = 2; 1 h post-reperfusion, n = 3; 2 h post-reperfusion, n = 3) were used. Note that lactate was not injected in any of these animals. At the set time-points after ischaemia-reperfusion, mice were sacrificed by intraperitoneal injection of 150 mg/kg sodium pentobarbital and intracardially perfused with 4% paraformaldehyde in phosphate buffer at pH 7.4. Dissected brains were post-fixed overnight, cryoprotected in 30% sucrose and cut as 30 μm cryostat coronal sections. Free-floating sections were blocked in 0.5% casein and then incubated overnight at 4 °C with primary antibodies in 0.1% Triton X-100-PBS-1% BSA, rinsed in PBS and incubated with Alexa Fluor-coupled secondary antibodies (ThermoFisher-Molecular Probes) and DAPI for nuclear counterstaining one hour at room temperature. Slices were finally rinsed in PBS and mounted in FluorSave™ (Calbiochem). The antibodies used were: anti-Microtubule Associated Protein-2 (MAP-2) mouse monoclonal antibody (Millipore #MAB3418, 1:400); anti-Platelet Endothelial Cell Adhesion Molecule-1 (PECAM-1, also known as CD31) rat monoclonal antibody (BD Pharmingen #550274, 1:100); anti-Monocarboxylate Transporter 4 (MCT4) rabbit polyclonal antibody (Santa Cruz Biotechnology #sc-50329, 1:500); anti-Monocarboxylate Transporter 1 (MCT1) and anti-Monocarboxylate Transporter 2 (MCT2) rabbit polyclonal antibodies^46^ were both used at a 1:500 dilution and were a kind gift of Prof. Luc Pellerin. Three z-stack images (9–11 images/stack, z = 1.5 μm) per section were acquired with a TCS SP5 RS – DM6000 confocal scanning microscope (Leica, Germany) at 20 x magnification (HC PL Fluotar, NA 0.5, air). Representative confocal images are displayed as maximal z-projections. Images were processed with Fiji/ImageJ software (NIH, USA) and Adobe Photoshop CS5 (Adobe, USA).

### Statistical analysis

We performed statistical analyses using the OriginPro 2016 software. One-way analysis of variance (One-way ANOVA) was used followed by Tukey’s test. A *p* value of 0.05 was considered significant. All data are presented as means ± standard deviation unless otherwise stated. For this feasibility study, we selected the group size based on our previous experience with this model^15,20,47^. We could not be blind to the measurements due to the imaging methodologies employed. Data can be accessed on request from the corresponding author.

### Ethical approval and informed consent

All experimental protocols were approved by the Vaud Cantonal Veterinary Office (VD2017.5). Experiments were carried out in accordance with the Swiss Animal Protection Act and the ARRIVE guidelines.

## Results

**Figure 1:**
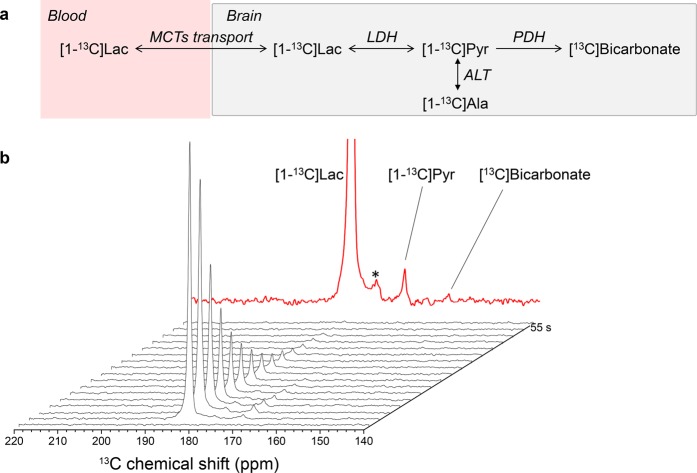
(**a**) Simplified scheme of the cerebral metabolism of HP [1-^13^C] lactate. (**b**) Dynamic ^13^C MRS spectra measured in the head of an MCAO mouse following the injection of HP [1-^13^C] lactate (183.5 ppm). The transfer of the label to [1-^13^C] pyruvate at 171.5 ppm can be readily detected. In the sum of the spectra shown in the top panel (red), the peak of ^13^C bicarbonate becomes visible at 161.5 ppm. The peak designated by (*) at 176 ppm results from chemical impurities that overlap with the expected [1-^13^C] alanine peak.

In Fig. 1, we show typical *in vivo* HP ^13^C MRS spectra from the brain of a single animal after MCAO surgery acquired 1 h post-reperfusion. The infusion of HP [1-^13^C] lactate leads to labelling of the pyruvate pool as mediated by lactate dehydrogenase (LDH). [1-^13^C] pyruvate is further metabolised to produce ^13^C labelled bicarbonate; however, due to limited signal-to-noise ratio (SNR) we could only clearly identify the bicarbonate signal in the sum spectra in 9 out of the 15 animals that received HP [1-^13^C] lactate. Chemical impurities at 176 ppm overlap with the expected [1-^13^C] alanine peak, preventing reliable quantification or dynamic interpretation of this metabolite. Pyruvate hydrate, which represents <8% of pyruvate at physiological conditions^32^ (as it depended on the pH and temperature^48^), was not detectable.

**Figure 2:**
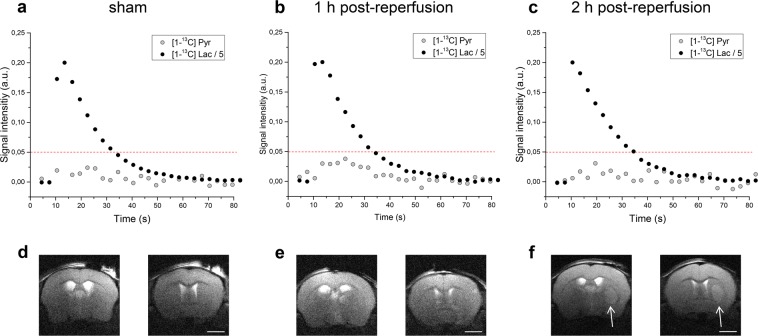
Representative *in vivo* time courses of [1-^13^C] lactate (black) and its metabolic product [1-^13^C] pyruvate (grey) in the brain of sham operated mice (**a**) and of mice at different times after ischaemia onset **(b**,**c**). Each time course was normalised to the respective maximal lactate signal. The red dotted line was added to highlight the differences in [1-^13^C] pyruvate labelling. The corresponding T_2W_ axial images of the brains of sham operated mice (**d**) and mice at different time points after ischaemia onset (**e**,**f**) are presented at the bottom of each time course. While a clear lesion can be detected 2 h post-reperfusion (**f**, white arrow), the morphological modifications are still obscured at 1 h post-reperfusion (**e**). Images acquired ca. 5 min before the infusion of HP [1-^13^C] lactate, showing two axial slices out of 14 one mm thickness slices. Other experimental parameters: effective echo time and repetition time: TE_eff_/TR = 52/4000 ms, 2 scans, 18 mm × 18 mm FOV with matrix size 256 × 256. Scalebar: 2 mm.

We quantified the AUC of the [1-^13^C] lactate and [1-^13^C] pyruvate signals to obtain the typical metabolic time course (Fig. 2). We observed larger [1-^13^C] pyruvate signals in 1 h post-reperfusion injected animals compared to 2 h post-reperfusion and sham (Fig. 2). 37.5 s after the beginning of HP [1-^13^C] lactate infusion, we barely detected pyruvate signals, and so we calculated pyruvate-to-lactate ratios (PLR) and bicarbonate-to-lactate ratios (BLR) from the spectra acquired during the first 37.5 s of the experiment. The corresponding anatomical T_2W_ images presented at the bottom of each time course illustrate that at 1 h post-reperfusion slight morphological modifications can be depicted, whereas 2 h post-reperfusion the characteristic striatal lesion following MCAO is readily visible. As expected, images of sham animals show no lesion.

**Figure 3:**
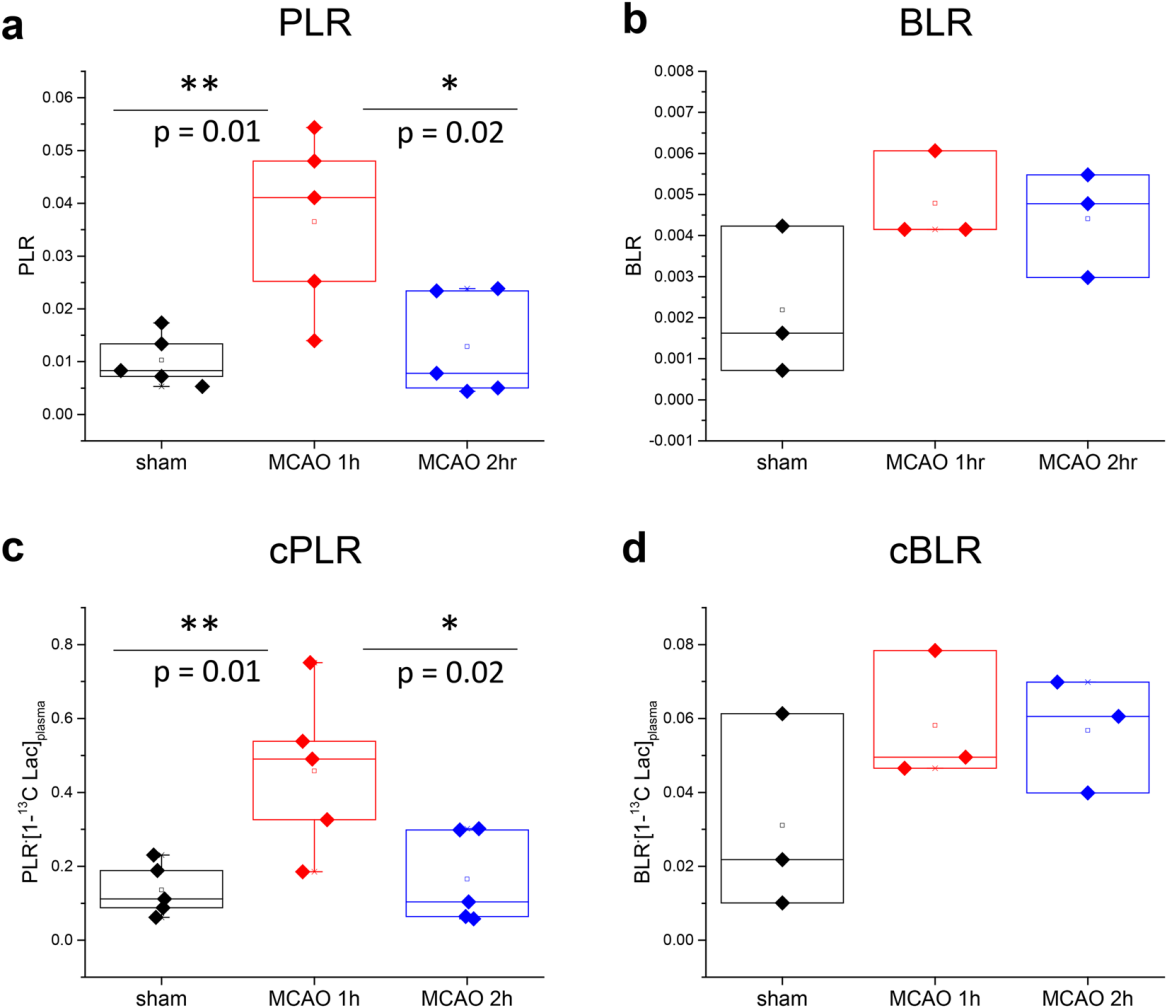
(**a**,**c**) Pyruvate-to-lactate ratios (PLR) and ratios corrected for [1-^13^C] lactate concentration in blood at the time of injection (cPLR), overlaid with individual data points. One-way ANOVA test indicates significant statistical difference (p < 0.05) between both PLR and cPLR 1 h post-reperfusion (red) and sham (black) and 2 h post-reperfusion (blue). The average values for cPLR were 0.46 ± 0.21, 0.17 ± 0.12 and 0.14 ± 0.07 in MCAO mice 1 h and 2 h post-reperfusion and sham respectively. (**b,d**) Bicarbonate-to-lactate ratios (BLR) and ratios corrected for [1-^13^C] lactate concentration in blood at the time of injection (cBLR) overlaid with individual data points showing a trend for increased BLR and cBLR in MCAO mice 1 h (red) and 2 h post-reperfusion (blue) compared to sham (black). The average values for cBLR were 0.058 ± 0.018, 0.057 ± 0.015 and 0.031 ± 0.027 in MCAO mice 1 h and 2 h post-reperfusion and sham respectively.

PLR and cPLR were significantly higher at 1 h post-reperfusion than 2 h post-reperfusion or in sham (p < 0.05). A trend to larger BLR and cBLR in animals experiencing MCAO surgery compared to sham animals was observed, however, no significant differences were found between the groups (Fig. 3).

**Figure 4:**
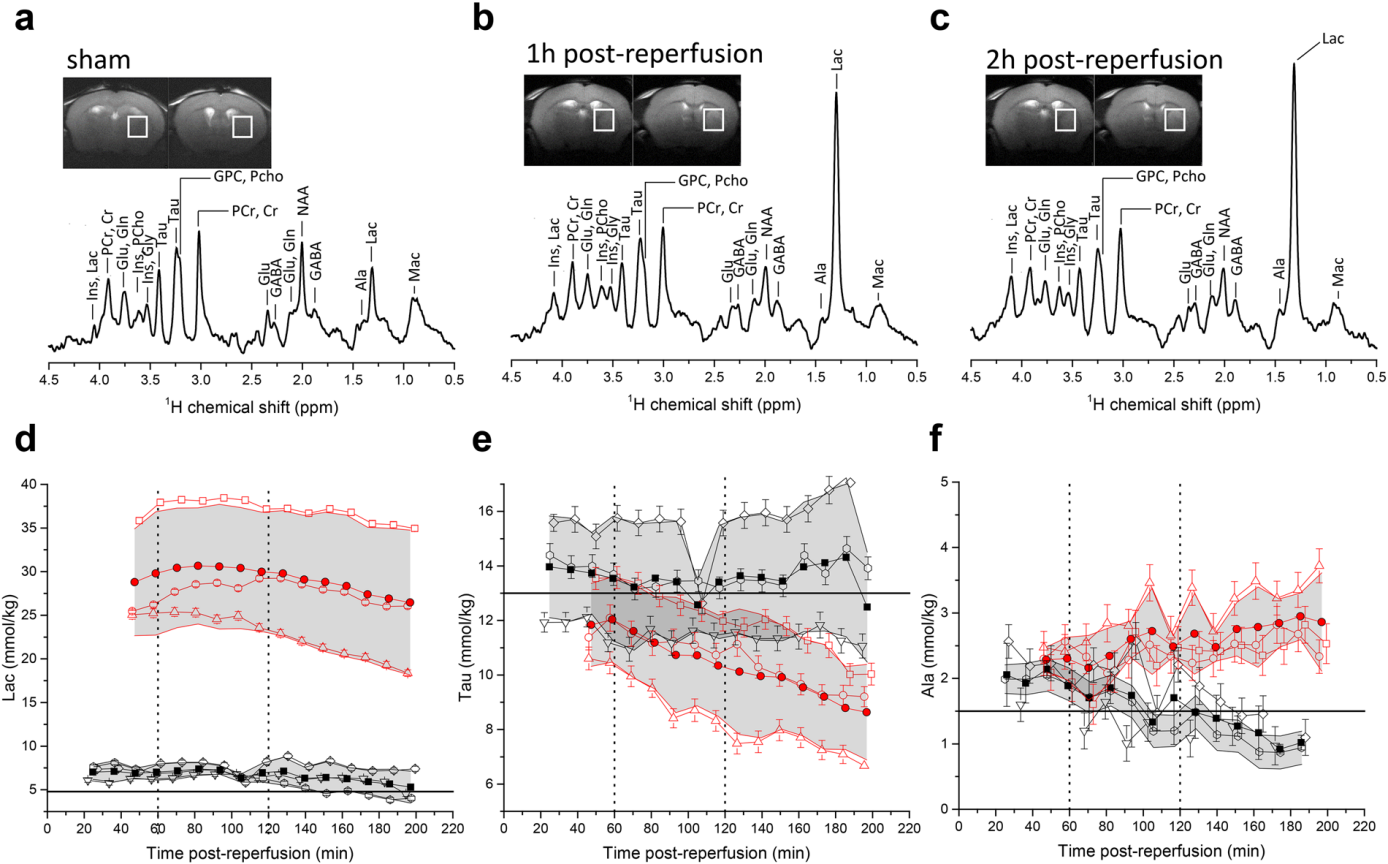
(**a–c**) Representative ^1^H spectra and voxel position in the striatum of sham mice and MCAO mice 1 h and 2 h post-reperfusion. (**d–f**) Time course of selected metabolites after 30 min MCAO surgery (red) and sham operated mice (black); time zero indicates the beginning of reperfusion. Open symbols are individual animals, with Cramer-Rao lower bounds (CRLB) of the LC-model quantification as error bars. Full red circles are the average of all three MCAO animals and full black squares are the average of all three shams -with the grey area representing the corresponding standard deviation. Horizontal lines that designate the metabolite concentration in healthy 8-week C57BL6/J mice as reported by Tkac *et al*.^49^ were added to guide the eye on the dynamic evolution of the metabolite concentrations. Vertical lines indicate the times at which HP lactate was injected in the HP ^13^C MRS measurements.

Representative proton spectra acquired in the striatum of sham operated and MCAO operated mice 1 h and 2 h post-reperfusion together with the dynamic evolution of several brain metabolites showing changes compared to baseline values^49^ in the first 200 minutes post-reperfusion are presented in Fig. 4. Data acquisition started as early as 20 min post-surgery in sham animals and 40 min post-reperfusion in MCAO mice. The ischaemic insult led to a large increase in lactate concentrations that reached a maximum value between 60 and 120 min post-reperfusion and then steadily decreased (Fig. 4d). The putative osmoregulator taurine (Tau) steadily decreased (Fig. 4e). Alanine (Ala) concentrations steadily increased in MCAO mice and steadily decreased in sham mice (Fig. 4f). These changes show similar trends to those reported in MCA occluded mice 180 min post-reperfusion^20^. The concentration of other metabolites quantified by the LC-model as well as the direction of significant changes are presented in Table 1.

**Table 1:** Neurochemical profiles in the striatum of sham operated mice and after 30 min of transient ischaemia.

metabolites	Sham (3)	~ 1-h (3)	Changes at ~1-h	~2-h (3)	Changes at ~2-h
Ala	2.0 ± 0.5	2.3 ± 0.3		2.6 ± 0.5	
Cr	4.5 ± 0.2	4.5 ± 0.2		4.2 ± 0.3	
PCr	4.3 ± 0.2	2.9 ± 0.5	↓ **	2.9 ± 0.4	↓ **
Cr+PCr	8.8 ± 0.3	7.4 ± 0.5	↓ *	7.2 ± 0.6	↓ **
PCr/Cr	0.95 ± 0.06	0.64 ± 0.12	↓ **	0.70 ± 0.07	↓ *
GABA	2.3 ± 0.2	3.4 ± 0.1	↑ **	3.2 ± 0.5	↑ *
Gln	3.1 ± 0.3	3.1 ± 0.3		3.0 ± 0.6	
Glu	7.0 ± 0.3	4.8 ± 0.6	↓ ***	4.2 ± 0.2	↓ ****
Glu+Gln	10.1 ± 0.4	8.0 ± 0.9	↓ *	7.2 ± 0.7	↓ ***
Gln/Glu	0.44 ± 0.04	0.65 ± 0.01	↑ p = 0.06	0.70 ± 0.15	↑ *
GSH	1.5 ± 0.3	1.3 ± 0.5		0.9 ± 0.3	
Gly	1.6 ± 0.3	2.4 ± 0.5		2.2 ± 0.4	
*myo-*Ins	5.9 ± 0.3	5.0 ± 0.6		4.3 ± 0.8	↓ *
*Myo-*Ino + Gly	7.4 ± 0.3	7.4 ± 0.1		6.5 ± 0.7	↓ p = 0.08
Lac	7.0 ± 0.8	29.2 ± 6.9	↑ ***	28.9 ± 6.8	↑ ***
NAA	6.7 ± 0.4	4.1 ± 0.4	↓ ****	3.8 ± 0.5	↓ ****
NAA + NAAG	6.9 ± 0.4	4.1 ± 0.3	↓ ****	3.9 ± 0.5	↓ ****
Tau	14.0 ± 1.9	11.6 ± 1.9		9.8 ± 2.3	
PE	2.9 ± 0.6	1.5 ± 0.2	↓ *	1.3 ± 0.4	↓ **
Mac	1.9 ± 0.1	1.2 ± 0.1	↓ ***	1.2 ± 0.1	↓ ****
GPC + PCho	1.10 ± 0.04	1.12 ± 0.18		1.03 ± 0.25	

Ala, alanine; Cr, creatine, PCr, phosphocreatine; GABA, γ-aminobutyric acid; Gln, glutamine; Glu, glutamate; GSH, glutathione; Gly, glycine; *myo-*Ins, myo-inositol; Lac, lactate; NAA, N-acetyl-aspartate; NAAG, N-acetyl-aspartate-glutamate; Tau, taurine; PE, phosphatidylethanolamine; Mac, macromolecule; GPC, glycerophosphocholine; PCho, phosphocholine. All concentrations, except ratios, are in mmol/kg. Number of animals is indicated in parentheses. Only metabolites  with CRLB of less than 45% in all animals are presented. The changes are calculated compared to sham and displayed with specific oriented arrows (‘↓’ as decrease and ‘↑’ as increase). The number of ‘*’ represent statistically significant levels of *p* = 0.05, 0.02, 0.01 and 0.001.

**Figure 5:**
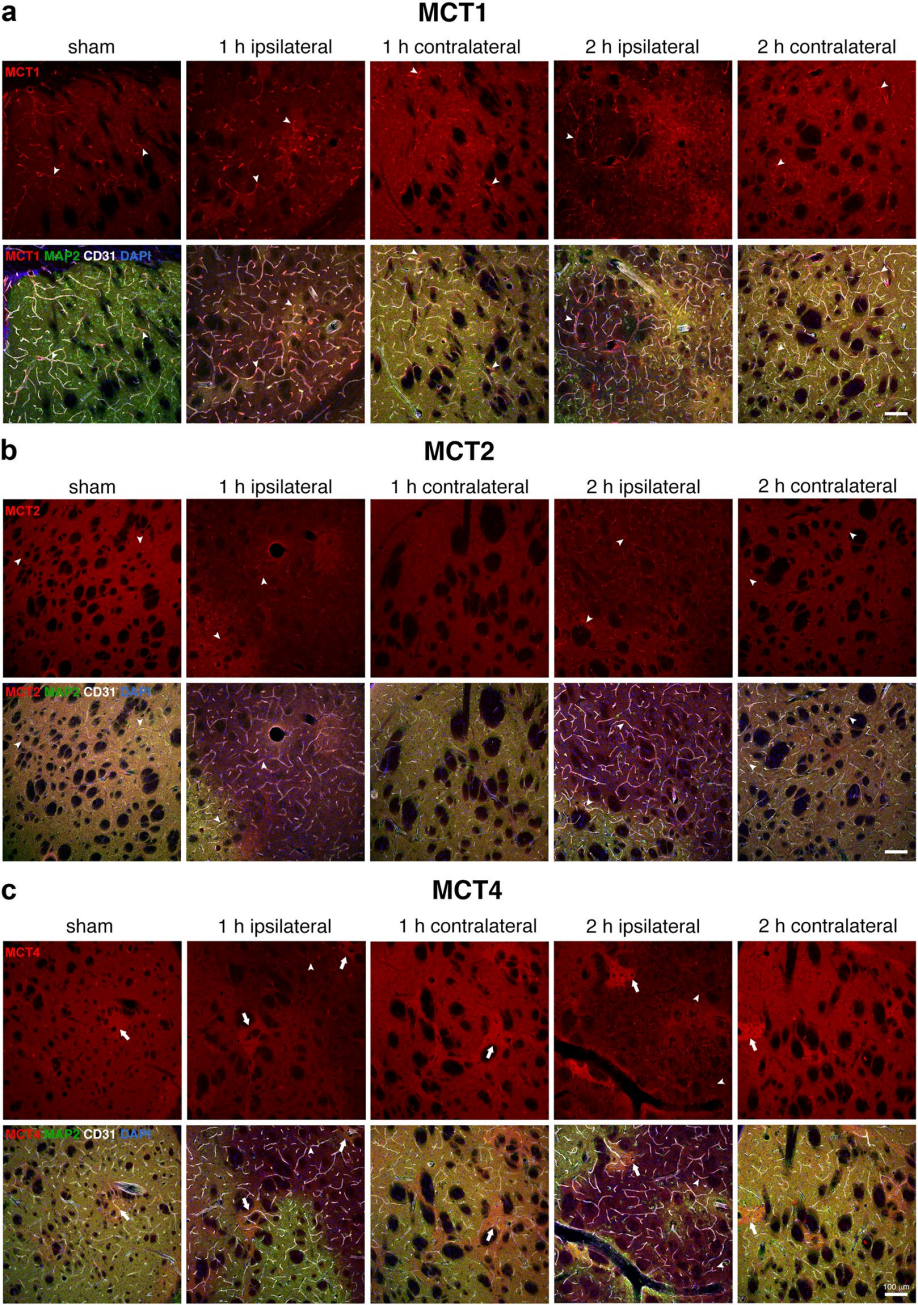
Changes in MCT expression patterns after ischaemia-reperfusion. The images in red in the upper panels show the expression of lactate transporters MCT1 (**a**), MCT2 (**b**) and MCT4 (**c**) in the striatum of sham animals and in the striatum ipsilateral and contralateral to the lesion of MCAO mice at 1 h or 2 h post-reperfusion. The lower panels show the merged images of the triple immunolabelling of the MCTs (red) with the neuronal marker MAP-2 (green) to identify the lesion area (i.e. loss of MAP-2 staining), the endothelial cell marker CD31 to identify blood vessels (grey) and the nuclear counterstaining (DAPI, blue). Arrowheads indicate blood vessels. Arrows in (**c**) indicate patches of high MCT4 expression. Scalebar: 100 μm.

In the brain, monocarboxylate transporters (MCTs) 1, 2 and 4 mediate bidirectional transport of lactate across cell membranes by diffusional, saturable symport with protons. In agreement to what has been previously reported^47^, we observed changes in the distribution of MCTs after transient MCAO. Whereas in sham animals intermediate affinity MCT1 (Km_lactate_ 3.5–10 mM, Fig. 5a) is mainly expressed in endothelial cells and brain parenchyma^46^, at 1 h after reperfusion the MCT1 signal is stronger in blood vessels and in areas where neurons are still present, while it decreases in areas of neuronal loss. 2 h after reperfusion, MCT1 expression is still prominent in vessels and in surviving neurons. The high-affinity transporter MCT2 (Km_lactate_ 0.5–0.75 mM, Fig. 5b) is expressed strongly in neurons and weakly around blood vessels in sham animals^46^. At 1 h post-reperfusion and even more intensely at 2 h post-reperfusion, we observe enhanced expression of MCT2 around blood vessels. The MCT2 signal is decreased in damaged areas of the striatum at both time-points, possibly due to the neuronal loss. The low-affinity, mostly astrocytic, lactate transporter MCT4 (Km_lactate_ 22–28 mM Fig. 5c)^50^ is distributed unevenly in the striatum of sham mice, with some patches of intense expression. Similar to MCT1 and MCT2, there is an overall decrease in expression of MCT4 in areas of neuronal loss, both 1 h and 2 h post-reperfusion. Especially 2 h after reperfusion, we observe MCT4 staining in small blood vessels in the lesion areas. The patches of stronger MCT4 signal observed in sham mice are still present 1 h and 2 h post-reperfusion. Changes in MCTs expression and distribution extend to the contralateral hemisphere, where they show similar patterns to those observed in areas of the ipsilateral side with surviving neurons.

## Discussion

This study demonstrates the feasibility of measuring in real-time *in vivo* metabolism of the neuroprotective agent [1-^13^C] lactate, administered at a therapeutic dose after a transient focal cerebral ischaemia using HP ^13^C MRS. We detected LDH mediated transfer^51^ of the ^13^C label from [1-^13^C] lactate to [1-^13^C] pyruvate, which is further transferred to ^13^C bicarbonate via PDH activity. We found a significantly higher cPLR at 1 h post-reperfusion compared to both 2 h post-reperfusion and sham animals. In addition, we observed a trend towards a higher cBLR in animals after MCAO surgery compared to sham animals, indicating that pyruvate is driven towards the TCA cycle to be metabolised (Fig. 3). Similar differences were observed for the uncorrected ratios. These findings imply that the ratios depend on the time elapsed between ischaemia onset and the beginning of reperfusion, probably reflecting the evolving metabolic reprogramming and energetic demands after the ischemic insult. Our global measurement of the entire brain indicates a significant difference in the cerebral metabolism of the exogenous HP [1-^13^C] lactate, however, at this stage we cannot identify whether it is arising from the lesion, penumbra, or remote brain area. A further step will be to localise the cPLR signals, for instance by imaging techniques, thereby mapping where the metabolic conversions take place, to understand the underlying metabolic condition of tissue in ischaemia. The SNR obtained with our current set up and HP sample formulation (in particular the chosen free radical) is too limited to perform signal localisation at a relevant spatial resolution. Improved SNR and localisation could then lead to the use of HP [1-^13^C] lactate as a molecular imaging biosensor in ischaemic stroke patients after endovascular clot removal, keeping in mind that lactate is known to improve the neurological outcome in preclinical studies^15^.

The neurochemical profiles of the ischaemic lesion area quantified from the ^1^H MRS spectra and their evolution after insult at the two HP [1-^13^C] lactate injection time points tested (i.e. 1 h and 2 h post-reperfusion) indicate differences in metabolite concentrations and, thus, suggest a change in the metabolic demand of the tissue at the two time points. Endogenous lactate concentrations at both 1 h and 2 h post-reperfusion were extremely elevated compared to sham and showed similar average concentrations. However, at 1 h post-reperfusion, the lactate concentration is nearly at its maximum value, while at 2 h post-reperfusion, lactate concentration is decreasing, indicating that endogenous lactate is either being consumed by the tissue or eliminated. This difference suggesting a shift in the metabolic dynamics of lactate at 1 h and 2 h may be related to the dissimilarity in response to the HP [1-^13^C] lactate bolus. Interestingly, previous findings from our lab have shown divergence in the neuroprotective effect of lactate when administered at different time points after stroke^15^. Lactate administered 1 h after ischaemia onset (and immediately post-reperfusion) decreased lesion size and ameliorated the neurological outcome. However, administration 2 h after onset (i.e. 1 h post-reperfusion) had no effect on the lesion size, but a stronger improvement in neurological outcome.

It is important to highlight that ^1^H MRS spectra were collected in a single voxel located within the striatal lesion, while the HP ^13^C MRS, following the bolus of [1-^13^C] lactate was detected over the entire brain. To better investigate the relationship between the neurochemical profile and the metabolic response to the HP [1-^13^C] lactate injection, it will be of high interest to improve the sensitivity of the HP [1-^13^C] lactate experiment to enhance the spatial resolution in order to localise and delimit the observed effect. Further improvement in the HP sample formulation, for instance using more efficient polarising agents (e.g. trityl), would allow enhancement of the initial polarisation level of injected [1-^13^C] lactate, and therefore increase the SNR of the entire measurement, in particular for detecting [^13^C] bicarbonate. Moreover, higher sensitivity would potentially enable us to quantify the kinetics of the transfer of the ^13^C labelling from HP [1-^13^C] lactate to other metabolites through biochemical reactions.

For the cerebral conversion of HP [1-^13^C] lactate, the substrate must first cross the BBB via MCTs before being converted to [1-^13^C] pyruvate by the LDH enzyme. It has been reported that in the healthy brain the BBB limits the observation of cerebral metabolism of HP monocarboxylic acids^34,52–54^, and that the transfer of the label to pyruvate is directly related to the endogenous pool size^51^. We found that at the HP [1-^13^C] lactate injection time points MCTs expression and distribution were altered in both brain hemispheres. MCT1, MCT2 and MCT4 expression was increased in blood vessels and MCT1 and MCT4 expression was increased in living neurons, perhaps enabling more lactate to be globally delivered to the tissue and resulting in a larger concentration of HP [1-^13^C] lactate in the brain compartment that is rapidly exchanged to [1-^13^C] pyruvate in cells with high energetic demand. This might be the dominant effect at 1 h post-reperfusion. However, the difference in the response to the [1-^13^C] lactate bolus 1 h and 2 h post-reperfusion is probably related to changes in lactate metabolism, as suggested by the differences in the neurochemical profiles. Further investigations are necessary to clarify the mechanisms in our system.

In this proof-of-concept study, we demonstrate the metabolism of exogenous HP [1-^13^C] lactate in the mouse brain in real time and *in vivo* after transient cerebral ischaemia and its relation to the time elapsed after reperfusion. The mouse transient MCAO with suture withdrawal at the end of the ischaemia is a realistic model of the clinical situation with intervention of clot removal. HP [1-^13^C] lactate has potential as a new molecular imaging biosensor after stroke, similar to HP [1-^13^C] pyruvate to characterise the damage as well as differences between the penumbra, necrotic core lesion and intact regions^31^. In addition, as lactate was shown to improve neurological outcome, administration could contribute to a better post-ischaemic recovery^15,16,18^. Moreover, further application of ^13^C MRS of HP lactate to study cerebral metabolism in the intact brain and in MCAO animal models may help clarify the mechanism of lactate neuroprotection and elucidate lactate’s proposed dual mode of action as energetic substrate and signalling agent^17^.

## Conclusion

HP [1-^13^C] lactate metabolism can be measured in a transient MCAO mouse stroke model after i.v. injection: injected lactate reaches the brain very rapidly and gets converted into pyruvate and CO_2_. Global HP ^13^C MRS measurements demonstrate time-dependent lactate metabolism in the first two hours after reperfusion, suggesting modification in the transport and/or cerebral metabolism.

## Supplementary information

Supplementary information.

## Data Availability

All data is available from the authors upon reasonable request.
