# Supplementary material for: Evaluating the potential of hyperpolarised [1-13C] L-lactate as a neuroprotectant metabolic biosensor for stroke
Source: Sci Rep. 2020 Mar 26;10:5507. doi: 10.1038/s41598-020-62319-x (PMC7099080; doi:10.1038/s41598-020-62319-x)
Supplement: Supplementary file 1 — Supplementary information. [file 41598_2020_62319_MOESM1_ESM.docx]

Supporting information

**Evaluating the potential of hyperpolarised [1-^13^C] L-lactate as a neuroprotectant metabolic biosensor for stroke**

Jean-Noël Hyacinthe, Lara Buscemi, Thanh Phong Lê, Mario Lepore, Lorenz Hirt, and Mor Mishkovsky


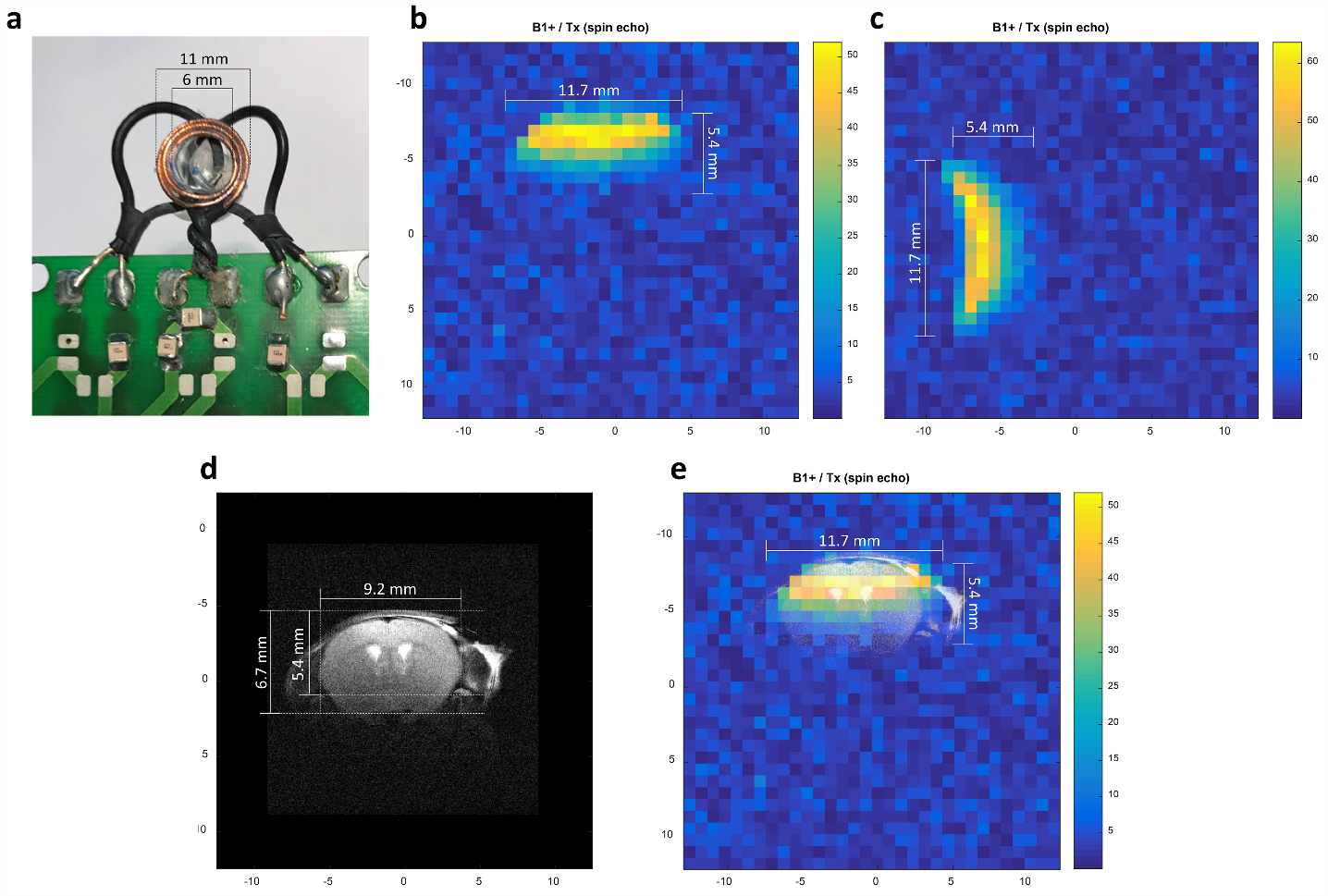


**S1: Surface coil specifications**: (a) Photo of the coil employed in this study. The ^13^C three loops surface coil is of 6 mm inner and 11 mm outer diameters. The butterfly ^1^H surface coil is composed by two loops of 13 mm inner and 15 mm diameters. (b) Axial and (c) sagittal B_1_^+^ maps were acquired on a cylindrical phantom (1.35 cm outer and 1.2 cm inner diameters, and 2 cm length), containing a 350 mM solution of sodium [1-^13^C] acetate and 10 mM Gadovist contrast agent leading to ^13^C T_1_ of 825 ± 16 ms. Acquisition parameters included: FOV of 25 mm by 25 mm, matrix size of 32 x 32, 1 slice of 1 mm thickness, TR = 5 s, TE = 13.06 ms, 2 dummy scans and 64 repetitions. (d) Typical T_2_W image acquired on sham operated mouse, acquired with FOV of 18 mm by 18 mm and matrix size 256 x 256, and presented in the same FOV of the B_1_ map. (e) Overlay of the 13C B_1_^+^ map on the ^1^H anatomical T_2_W image of the mouse brain.
